# Hormone Targets for the Treatment of Sleep Disorders in Postmenopausal Women with Schizophrenia: A Narrative Review

**DOI:** 10.3390/clockssleep4010007

**Published:** 2022-02-15

**Authors:** Alexandre González-Rodríguez, José Haba-Rubio, Judith Usall, Mentxu Natividad, Virginia Soria, Javier Labad, José A. Monreal

**Affiliations:** 1 Department of Mental Health, Mutua Terrassa University Hospital, University of Barcelona, 08221 Terrassa, Spain; mnatividad@mutuaterrassa.cat; 2 Centre for Investigation and Research in Sleep (CIRS), Centre Hospitalier Universitaire Vaudois, 1011 Lausanne, Switzerland; Jose.Haba-Rubio@chuv.ch; 3 Parc Sanitari Sant Joan de Déu, Sant Boi Llobregat, 08830 Barcelona, Spain; judit.usall@sjd.es; 4 Department of Psychiatry, Bellvitge University Hospital, Bellvitge Biomedical Research Institute (IDIBELL), Neurosciences Group—Psychiatry and Mental Health, CIBERSAM, University of Barcelona, 08907 L’Hospitalet de Llobregat, Spain; vsoria@bellvitgehospital.cat; 5 Department of Mental Health, Consorci Sanitari del Maresme, Fundació Parc Taulí, CIBERSAM, 08340 Mataró, Spain; jlabad@csdm.cat; 6 Neurosciences Institute, Universitat Autònoma de Barcelona, CIBERSAM, 08221 Terrassa, Spain; jamonreal@mutuaterrassa.cat

**Keywords:** schizophrenia, delusional disorder, sleep, insomnia, treatment

## Abstract

While the early identification of insomnia in patients with schizophrenia is of clinical relevance, the use of specific compounds to treat insomnia has been studied less in postmenopausal women with schizophrenia. We aimed to explore the effects of melatonin, sex hormones, and raloxifene for the treatment of insomnia in these populations. Although melatonin treatment improved the quality and efficiency of the sleep of patients with schizophrenia, few studies have explored its use in postmenopausal women with schizophrenia. The estrogen and progesterone pathways are dysregulated in major psychiatric disorders, such as in schizophrenia. While, in the context of menopause, a high testosterone-to-estradiol ratio is associated with higher frequencies of depressive symptoms, the effects of estradiol and other sex hormones on sleep disorders in postmenopausal women with schizophrenia has not been sufficiently investigated. Raloxifene, a selective estrogen receptor modulator, has shown positive effects on sleep disorders in postmenopausal women. Future studies should investigate the effectiveness of hormonal compounds on insomnia in postmenopausal women with schizophrenia.

## 1. Introduction

Schizophrenia is a severe mental illness, with an estimated prevalence of 1% in the global population [1], and a high burden of disease [2]. Schizophrenia is widely characterized by the presence of positive symptoms, which include delusions, hallucinations, and disorganized behavior. The negative symptoms mainly include affective flattening, apathy, and social withdrawal, as well as cognitive deficits and nonprominent affective symptoms [3].

Sleep disturbances have not classically been considered a diagnostic criterion for schizophrenia; however, the vast majority of studies support the notion that patients with schizophrenia suffer from sleep disturbances in at least 80% of cases [4]. In a recent study by Reeve et al., which included outpatients with nonaffective psychosis, 80% of the patients suffered from at least one sleep disorder, with insomnia and nightmare disorder being the most common [5].

In particular, some researchers report that over half of the patients presenting persecutory delusions may present moderate or severe insomnia [4], which may have clinical consequences. For instance, patients with schizophrenia who suffer from sleep disorders may present higher frequencies of psychotic experiences, depressive symptoms, and anxiety, and lower quality of life [5]. Additionally, a naturalistic longitudinal study in outpatients with schizophrenia spectrum disorders highlights that patients with insomnia may show increased incidences of suicide attempts during the follow-up, and that the comorbidity between nightmare complaints and insomnia may increase the risk of suicide attempts [6]. The authors conclude that sleep disturbances are common in patients with schizophrenia and are associated with the risk of suicide, a fact that highlights the need for more clinical attention, as well as the need for an urgent intervention into sleep disturbances in patients with schizophrenia [6]. This is in agreement with a recent study by Miller et al., that recruited 108 patients with nonaffective psychosis, including schizophrenic individuals. The authors explored the association between insomnia, suicidal ideation, and the suicide-attempt histories of patients [7]. The results were quite interesting, as the patients with severe insomnia were more likely to have lifetime histories of suicide attempts than patients without insomnia, which suggests, once again, that an assessment of insomnia may be a marker of suicide risk and the severity of the symptoms.

Given the clinical relevance of the identification of insomnia in patients with schizophrenia, an adequate treatment is mandatory. Stummer et al. [8] reviewed the most recent works of the last two decades on the treatments for insomnia in patients with schizophrenia, which include the use of melatonin, sodium oxybate, zopiclone or eszopiclone, and sedative antipsychotics [8]. This work confirms that there are few studies that focus on the pharmacological treatment options for insomnia in schizophrenia, and the majority of them that do suggest potential roles for melatonin, paliperidone, and eszopiclone. Few previous studies have investigated the roles of sex hormones in the treatment of insomnia in these populations.

Several hypotheses have been tested in order to elucidate the biological underpinnings of insomnia and other sleep disorders in patients with schizophrenia, and to address the potential targets of treatment. A review on the topic reports that the hyperactivity of the dopaminergic system, the dysfunction of the GABAergic system, and signaling in the sleep- and wake-promoting regions of the brain may be implicated [9], which suggests that multiple neurotransmitter systems are involved, and that several treatment options can be considered [9].

Genetic studies have revealed that variants in the promoter region of the melatonin receptor gene, MTNR1A, may be associated with schizophrenia and sleep disorders [10]. In an analysis of clinical phenotypes, rs2119882 of the MTNR1A gene was associated with insomnia in schizophrenia, but not with hypersomnia, which suggests that melatonin may be a potential target for patients with insomnia. Other studies that focus on the melatonin levels also confirm this potential association. In their sample of schizophrenia patients, Afonso et al. [11] found negative associations between the melatonin levels and the sleep latencies, the total sleep times, and the sleep efficiencies, in line with results from previous works [12], which suggests that the circadian patterns of melatonin secretion are altered in patients with schizophrenia. Insomnia has been associated with cognitive impairment in psychosis, and several trials have tested the potential effect of ramelteon—a melatonin receptor agonist—on the cognitive function of patients with schizophrenia, particularly in terms of learning and memory deficits [13]. However, few studies have focused on the effect of melatonin while attending to gender-specific differences.

On the other hand, a large amount of research indicates that there is a significant association between the sex hormone levels and the psychopathological symptoms in patients with schizophrenia, even in those patients with patterns of treatment response. Thomas et al. [14] investigated the influence of the serum hormones on the psychopathological symptoms of 45 women with schizophrenia, with a group-based trajectory-modeling design, using scales to assess the psychotic and mood symptoms. For the Positive and Negative Syndrome Scale (PANSS) scores, one subgroup had lower symptom severities, which were associated with the levels of follicle-stimulating hormone (FSH), dehydroepiandrosterone (DHEA), and luteinizing hormone (LH). A more recent study by the same group differentiated between two different groups according to the treatment response biomarkers [15]. The treatment response group was significantly negatively predicted by the estradiol blood levels, and the treatment nonresponse group showed a positive association with the FSH blood levels. More recently, Huang et al. [16] found serum progesterone levels to be negatively associated with the PANSS total scores, which suggests a correlation between the progesterone hormone levels and the disease severity of schizophrenia [16], which is seemingly in line with Gogos et al. [17]. In their review, the authors emphasize that estradiol and progesterone may be dysregulated in major psychiatric disorders, such as, for instance, schizophrenia.

During the progesterone phases of the menstrual cycle, the positive symptoms appear to improve, while, in the estrogen phases, the negative symptoms and the general psychopathological symptoms show significant improvements [18,19]. This is also in line with animal models of schizophrenia that assess the effects of progesterone, estradiol, and selective estrogen receptor modulators (SERMs) [20]. It was found that progesterone reduced the hyperactivity of the male and female dopamine transporters in knockout mice [21], and that SERMs prevented dopamine D1/D2-receptor-mediated disruptions in sensorimotor gating, which is similar to the effect of estradiol [22]. Few studies have investigated the association between sex hormones and psychopathology in postmenopausal women with schizophrenia [23].

The menopausal transition and the postmenopausal period are associated with a significant increase in sleep disorders, such as sleep apnea, insomnia, and restless leg syndrome [24]. Insomnia is the sleep disorder most commonly found in women, and it has been associated with negative impacts on healthcare, quality of life, and functionality [25]. Furthermore, sleep deprivation has been identified as a risk factor for cardiovascular disease, diabetes, obesity, and neurobehavioral disturbances. Some recent studies recommend that primary insomnia be preferentially treated with cognitive behavioral therapy or melatonin [25]. In fact, in healthy women, cognitive behavioral therapy represents the first line of treatment for insomnia in the general population [26], and the prolonged release of melatonin is recommended for women aged 55 years or older. However, to date, there are no studies that have correlated the sex hormone levels with insomnia and other sleep disturbances in women with schizophrenia. This is why an investigation of the effects of raloxifene—a SERM drug—in the treatment of insomnia may be of interest in the context of schizophrenia, particularly in cases of postmenopausal women.

Thus, the main goal of this nonsystematic narrative review is to investigate the effects of melatonin, the sex hormones, and raloxifene for the treatment of insomnia on postmenopausal women with schizophrenia. The selected studies were chosen on the basis of the following review questions about postmenopausal women with schizophrenia:

(1) Does the treatment of melatonin improve insomnia in women with schizophrenia at the end of the reproductive lifespan?
(2) Does the use of estradiol, progesterone, and other sex hormones make a difference when treating insomnia?
(3) Does raloxifene improve insomnia in postmenopausal women with schizophrenia?

## 2. Methods

A nonsystematic narrative review was performed that was based on electronic searches of the PubMed and ClinicalTrials.gov databases, over the last two decades (2000–2021 October), for English, Spanish, German, or French papers that address our three main questions. Papers were included only if they were focused on the treatment effects of melatonin, raloxifene, progesterone, estradiol, or other sexual hormones, on sleep disorders in patients with schizophrenia, and particularly in postmenopausal women.

In order to start our search strategy in PubMed, we initially used the following search strategy: (melatonin OR progesterone OR estradiol OR estrogens OR raloxifene) AND schizophrenia. We also searched Clinicaltrials.gov to find trials exploring the effects of melatonin, estradiol, progesterone, raloxifene, and other sex hormones on insomnia in postmenopausal women with schizophrenia. The following search strategy was performed: schizophrenia AND melatonin, schizophrenia AND estradiol, schizophrenia AND progesterone, schizophrenia AND raloxifene, schizophrenia AND testosterone, and schizophrenia AND sex hormones. Additionally, the reference lists from the included studies, as well as Google Scholar, were also evaluated in order to identify further studies.

**Figure 1:**
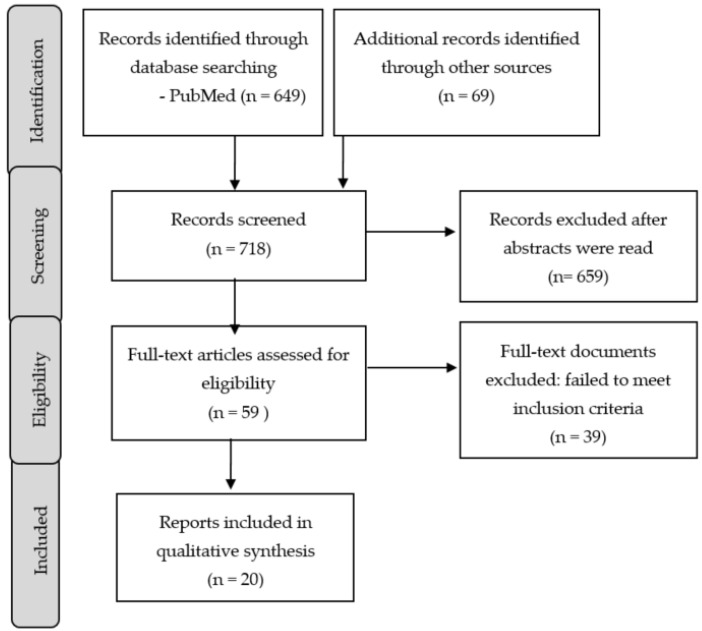
Flowchart for search leading to study inclusion.

The screening and selection processes were undertaken by two authors (A.G.-R. and J.A.-M.), who scanned all the titles and abstracts and subsequently selected the papers for the further scrutiny of their full texts. The inclusion criteria were as follows: (1) Meta-analyses and systematic reviews that followed PRISMA; (2) Randomized controlled trials; (3) Observational and prospective cohort studies; and (4) Studies focusing on the effects of melatonin, raloxifene, and the sex hormones on insomnia in postmenopausal women. Expert consensus by recognized scientific associations were also included for a further discussion of the findings reported in studies that focused on sleep in postmenopausal women with schizophrenia. The exclusion criteria were as follows: (1) Case reports; and (2) Studies not adequately describing the assessment measures used to evaluate insomnia. In cases where several papers refer to the same works, we included the publication containing the largest sample of patients that tested our questions. After the screening of all of the full-text documents that were accessible to the authors, a total of 20 records were included. Figure 1 represents the flowchart of all the included studies.

## 3. Results

### 3.1. Melatonin for the Treatment of Sleep Disorders in Postmenopausal Women with Schizophrenia

Sleep dysfunction has been widely recognized as a common clinical problem in people diagnosed with schizophrenia. Melatonin (N-acetyl-5-methoxytryptamine) is an endogenous hormone that is secreted in the pineal gland, and it has extensively been shown to have neuroprotective and antioxidant properties [27]. A double-blind placebo-controlled eight-week trial investigated the efficacy of ramelteon for the treatment of outpatients with schizophrenia [28]. Ramelteon is an agonist that acts solely on the melatonin MT(1) and MT(2) receptors [28]. The weights, the waist circumferences, the anthropometric measurements, and the metabolic markers of the patients were assessed. Although ramelteon did not improve the glucose metabolisms or inflammatory markers, the total cholesterol, and the cholesterol-to-high-density lipoprotein decreased, compared to the control group. In view of the potential effects of ramelteon, a melatonin agonist, several other studies have focused on its effects on clinical symptoms and insomnia.

Sahbaz et al., (2019) investigated the association between the serum melatonin concentrations and the cognitive impairment and circadian rhythms [29] in 47 patients with schizophrenia, and they compared them to 40 healthy controls. To evaluate the insomnia and the psychopathological symptoms, the authors used the Positive and Negative Syndrome Scale (PANSS), the Morningness–Eveningness Questionnaire (MEQ), and the Pittsburgh Sleep Quality Index (PSQI). After adjustments for age, sex, and body mass index, the patients with schizophrenia showed lower levels of melatonin compared to the healthy controls, which suggests that the melatonergic system should be investigated in future studies, and that the melatonin levels of patients receiving antipsychotics may be altered. Maiti et al. [30] confirmed these findings in their prospective cohort study: the urinary melatonin levels were found to be decreased in patients receiving haloperidol and risperidone.

Moreover, a systematic literature review focusing on clinical trials of patients with schizophrenia reveals that melatonin may have positive effects on sleep, the metabolic profile, and tardive dyskinesia [27]. This was also confirmed by Palagini et al. [31], who report that the administration of prolonged-release melatonin at 2–10 mg may be used for the treatment of insomnia in schizophrenia, particularly during acute phases [32].

A randomized rater-blinded clinical trial was conducted by Mishra et al. [33] to evaluate the effect of ramelteon—a melatonin receptor selective agonist—on the sleep and circadian rhythms of 120 patients with schizophrenia [33]. The patients were divided into two groups according to the presence of predominant positive or negative symptoms, and they were then randomized into the control groups receiving haloperidol or risperidone, or into the ramelteon groups. The serum melatonin was measured at baseline, as were the urinary melatonin and the sleep quality. After four weeks of treatment, the patients treated with ramelteon combined with antipsychotics showed an increase in the serum and urinary melatonin and improvements in the PANSS total scores, suggesting that ramelteon could reduce psychotic symptoms and improve sleep and circadian-rhythm disturbances in patients with schizophrenia; this is in line with previous studies. Suresh Kumar et al. [34] performed a randomized controlled trial to test whether two-week-long treatments of melatonin were effective at treating insomnia in patients with schizophrenia, compared to patients treated with placebos. Flexible doses of melatonin were administered (3–12 mg/night) to 20 outpatients with schizophrenia. A total of 20 other patients received placebos. A double-blind assessment of the sleep disturbances was conducted through the use of questionnaires, which were administered daily for the 15 days following the onset of the study. The patients receiving melatonin had increases in the quality of their sleep and the lengths of time that they slept. Furthermore, the patients receiving melatonin presented longer durations of sleep and daytime functioning compared to the control group, which suggests that, in particular cases, melatonin may be useful for treating insomnia in patients with schizophrenia. This finding is also presented in a previous study by Shamir et al. [35], who conducted a double-blind cross-over trial on 19 patients with schizophrenia who were randomized to melatonin at 2mg/day or a placebo. The urine melatonin levels were determined every three hours. For the sleep-disorder assessment, actigraphs were performed for three consecutive nights, at the end of each period. The treatment with melatonin improved the sleep efficiencies, compared to the placebo, and increased the sleep durations. There was no specific mention of the particular efficacy of melatonin on women with schizophrenia.

The effectiveness of prolonged-release melatonin for benzodiazepine discontinuation was also assessed in patients with schizophrenia and bipolar disorder in a randomized placebo-controlled blinded trial, with 24 weeks of follow-ups [36]. The patients were randomized to the following two groups: (1) A group treated with prolonged-release melatonin at 2 mg daily; or (2) A group treated with the placebo. The main outcome was the mean dose of benzodiazepines after 24 weeks of treatment. At the end of the follow-up period, the authors found that the patients receiving melatonin did not show any advantages compared to the control group. This suggests that prolonged-release melatonin did not facilitate benzodiazepine discontinuation in patients with schizophrenia.

In summary, melatonin treatment seems to improve the sleep quality and efficiency in patients with schizophrenia compared to patients taking placebos. There were no specific mentions of the use of melatonin in postmenopausal women with schizophrenia, which is a potential target population. Table 1 summarizes the main characteristics of the most representative studies that focus on the effectiveness of melatonin or melatonin agonists on insomnia in patients with schizophrenia.

### 3.2. Sex Hormones for the Treatment of Sleep Disorders in Postmenopausal Women with Schizophrenia

It has been reported that endogenous and exogenous hormone components interact with sleep regulation in men and women in adulthood [37]. Higher testosterone secretion has been found in men during sleep, and testosterone levels decrease as men age. In women, estrogen and progesterone exposure also seem to have effects on sleep regulation [37]. In premenopausal women, estrogen levels vary across the menstrual cycle, and variations have also been described during pregnancy and the postpartum periods. The effect of contraceptives is promising; however, few high-quality studies are available with respect to the effects of progestogens and estrogens for the treatment of insomnia in women [38]. Hormonal changes during the menopausal transition may have an impact on sleep, including in those women diagnosed with psychosis. We will revise the use of sex hormones for the treatment of sleep disturbances, particularly in women with schizophrenia, for menopause and beyond.

#### 3.2.1. Estradiol Use to Treat Sleep Disorders

There are animal studies that report that estrogens may influence sleep regulation [37]. In premenopausal women, the particular effect of estrogens on sleep during the menstrual cycle has been hypothesized, but the association has been not clearly elucidated. There are a large number of studies focusing on the menopausal transition and the postmenopausal periods. In fact, Kalleinen et al. [39] conducted a 10-year observational follow-up study that aimed to explore the changes in the sleep architecture during the menopausal transition in 57 premenopausal women. At the time of the inclusion of the patient in the study, and after 10 years of follow-up, a polysomnography (PSG) was performed, and the serum-follicle-stimulating hormone was determined. Although many studies have mentioned the sleep disruptions that can occur during the menopausal transition, the authors found that sleep disturbances were associated not only with menopause (and sex hormone changes), but likely also with increasing age.

Within the field of depression, controversial findings have also been reported with respect to sex hormones and sleep disorders. A systematic review by Morssinkhof et al. [40] explored research focused on the association between sex hormones, sleep, and depression. The authors found that patients with depression may suffer more frequently from sleep disorders; however, no statistically significant differences were found between the patients and the controls in terms of the endogenous hormone levels, which suggests that further research is required in order to draw future conclusions.

With all these questions in mind, we explored the potential association between estrogens and sleep disorders in women at the menopausal transition and after menopause, which are periods characterized by a decline in estrogen levels.

It is a well-established fact that estrogens are dysregulated in major psychiatric disorders, such as in schizophrenia. A review of the field reveals that estradiol has organizational and activational effects via genomic and nongenomic mechanisms [17]. In particular, animal models have been used to compare the effects of 17-beta-estradiol and the selective estrogen receptor modulators (raloxifene and tamoxifen) on the prepulse inhibitions in female rats [22]. Both raloxifene and estradiol were capable of preventing the dopamine D1/D2-receptor-mediated disruptions of sensorimotor gating, suggesting that estradiol and raloxifene may be considered as potential alternatives for the treatment of schizophrenia.

The effects of estradiol and other similar compounds on sleep disorders in postmenopausal women with schizophrenia have not been investigated. There are more studies available on the effects of selective estrogen receptor modulators (SERMs) in postmenopausal women with schizophrenia, which will be addressed in the following sections. Table 2 presents the hypothesis that estrogens are potential targets in the treatment of insomnia in postmenopausal women with schizophrenia.

#### 3.2.2. Progesterone Use to Treat Sleep Disorders

In a study by Huang et al. [16], the serum progesterone levels were negatively associated with psychotic symptoms, as they are measured by the PANSS total scores, which suggests a correlation between the hormone levels and the severity of the schizophrenia. Similar results have been described with regard to first-episode psychosis patients. Cai et al. [41] compared the neurosteroids in the pregnenolone pathways in controls and antipsychotic-naïve patients with schizophrenia, and they found that the alterations of the pregnenolone–progesterone–allopregnanolone pathways may be associated with the response to treatments. These findings open up the possibility for considering progesterone as a potential therapeutic agent for patients with schizophrenia, and particularly for women.

In postmenopausal women with schizophrenia, the effects of progesterone or other similar compounds have not been tested. Although brexanolone—a synthetic allopregnanolone—has been demonstrated to prevent depression-like behaviors in animal models, the psychotropic properties of progesterone have not been evaluated in postmenopausal schizophrenia [42].

#### 3.2.3. Testosterone and Dehydroepiandrosterone (as a Precursor of Testosterone) to Treat Sleep Disorders

The menopausal transition and postmenopausal stages in women are associated with an increased risk of sleep disturbances, which are partly explained by the influence of testosterone and the testosterone-to-estradiol ratio [43]. The most recent study investigates the effects of testosterone and the testosterone–estradiol ratio on the depressive symptoms, sleep quality, and vasomotor symptoms of 50 perimenopausal women without depression (aged 45–55 years). Menopause is characterized by a higher testosterone-to-estradiol ratio and is associated with higher frequencies of depressive symptoms [43]. In postmenopausal women with schizophrenia, similar findings have not been replicated. Further investigation is needed on targeting testosterone to treat postmenopausal women.

Dehydroepiandrosterone sulfate (DHEA-S) is mainly produced by the ovaries and the adrenal system. Dihydrotestosterone, a primarily peripheral product of testosterone metabolism, is also an androgen precursor in women. A study by Buoli et al. [18] investigated the association between progesterone and dehydroepiandrosterone sulfate in 89 drug-free men with schizophrenia, and DHEA-S was associated with higher probabilities of lifetime psychotic symptoms. The potential effects of DHEA-S on sleep disturbances in women with schizophrenia have not yet been investigated.

#### 3.2.4. Raloxifene Use to Treat Sleep Disorders

Raloxifene is a selective estrogen receptor modulator (SERM) that shows a high binding affinity to the estrogen receptor, and that acts as an estrogen agonist in bones and lipids, and as an antagonist in the uterus and breasts [44]. Furthermore, several animal studies have also pointed out that raloxifene may have an action on the central nervous system.

Natale et al. [45] conducted a case-control study with three months of follow-ups, with the main aim of investigating the effects of raloxifene (60 mg/day) on the sleep, affective symptoms, and cognitive functioning of 49 postmenopausal women. The authors found that raloxifene had no effect on the patients’ mood and cognitive symptoms, but that it improved their sleep by reducing the wakening episodes. This result is confirmed by Biri et al. [46], who conducted a comparison study that included 50 postmenopausal women treated with raloxifene, and a control group of 50 postmenopausal women without osteoporosis [46]. The Beck Depression Inventory (BDI) and the Insomnia Severity Index (ISI) were used to evaluate the depression and sleep disorders in postmenopausal women. Briefly, the ISI comprises seven items, which include the severity of sleep onset and maintenance, the satisfaction with the current sleep pattern, and the interference with daily functioning. The authors found that patients receiving raloxifene presented better-quality sleep.

The effect of adjunctive raloxifene for postmenopausal women with schizophrenia has been reported in recent meta-analyses [47,48]. Zhu et al. [47] conducted a meta-analysis of randomized double-blind placebo-controlled trials that examined the efficacy and safety of raloxifene in postmenopausal women suffering from schizophrenia. The women receiving raloxifene presented significant improvements in their positive and negative symptoms, including those general psychopathological symptoms that comprise anxiety and sleep symptoms. Wang et al., confirmed these findings [48].

While it has been extensively reported that raloxifene exerts a positive influence on sleep disturbances in healthy postmenopausal women, research that is focused on postmenopausal women with schizophrenia is scarce.

Table 3 presents the main findings on the effects of sex hormones and raloxifene on the treatment of insomnia in postmenopausal women with schizophrenia.

## 4. Discussion

This nonsystematic narrative review focuses on the following issues with regard to postmenopausal women with schizophrenia: In the first step, we tried to elucidate whether the treatment with melatonin is capable of improving insomnia in women with schizophrenia, at menopause and beyond. As a second objective, we investigated the use of estradiol, progesterone, and other sex hormones for the treatment of insomnia in these populations. Finally, we investigated whether raloxifene—a selective estrogen receptor modulator—may be a potential treatment for insomnia in postmenopausal women with schizophrenia, as it has been extensively explored to treat psychopathological symptoms.

The first question was addressed because several findings in animal models seem to indicate that melatonin may be useful for treating patients with schizophrenia. Melatonin treatment attenuated the schizophrenia-like symptoms in the mice and reduced the histopathological alterations [49]. Melatonin was particularly correlated with an attenuation of behavioral deficits and a reduction in the brain oxidative stress in a rodent model of schizophrenia [50], while other authors have reported contrary findings [51]. Furthermore, the coadministation of melatonin has been associated with improvements in the olanzapine-induced metabolic effects [52]. Studies in humans have confirmed some of the previous findings. A systematic review and meta-analysis of randomized controlled trials indicates that treatment with melatonin and melatonin agonists improves antipsychotic-induced metabolic syndrome [53], and that it is a good option for insomnia in schizophrenia [54]. All of these findings generated the hypothesis that melatonin treatment would be useful for patients with schizophrenia, especially for women in the postmenopausal stage.

In our review, we found that the serum melatonin concentrations were associated with cognitive impairment and the circadian rhythms [29] in patients with schizophrenia, and that the patients receiving haloperidol and risperidone presented lower urinary melatonin levels [30]. Treatment with ramelteon—a melatonin receptor selective agonist—increased the serum and urinary melatonin levels, and improved the psychotic symptoms [30,34]. Overall, we found that melatonin treatment improves the quality and efficiency of the sleep of patients with schizophrenia, compared to treatment with a placebo. However, there is a lack of studies exploring the effectiveness of melatonin or its derivates on insomnia in postmenopausal women with schizophrenia. Future studies should target this treatment towards this specific population.

It has been hypothesized that estradiol, progesterone, and other sexual hormones exert an effect on the central nervous system, and this has been evaluated in animal models of schizophrenia [21,22]. Furthermore, it has been suggested that estrogen replacement therapy has positive effects, but the clinical data are controversial. In the last few decades, several authors have reported that the administration of 17-beta-estradiol as an adjunctive treatment in ovariectomized rats may increase the antipsychotic efficacy in the animal model of psychosis during menopause [55]. It has been suggested that 17-beta-estradiol has antipsychotic activity as it has organizational and activational effects via genomic and nongenomic mechanisms [17]. Progesterone has been also negatively associated with psychotic symptoms [16], and, in the context of menopause (characterized by a higher testosterone-to-estradiol ratio), testosterone levels have been associated with higher frequencies of depressive symptoms [43]. However, in our review, we found that the effects of estradiol, progesterone, and other sex hormones have not been sufficiently investigated in the field of sleep disorders in postmenopausal women with schizophrenia.

The third question focuses on the use of selective estrogen receptor modulators (SERMs) in postmenopausal women with schizophrenia. The research on the use of SERMs has been restricted to the effects of raloxifene on the psychopathological symptoms and the cognitive domains [56,57]. Few studies report effects on sleep disturbances. In our review, we found that raloxifene does not have substantial effects on the mood and cognitive symptoms in healthy postmenopausal women; however, the evidence supports its beneficial effect on sleep disorders. Women receiving raloxifene showed better sleep quality compared to the control group [46]. This finding supports the hypothesis that asserts the positive effect of raloxifene with regard to insomnia on postmenopausal women with schizophrenia.

The use of estrogen modulators as adjunctive treatments has yielded promising results in the studies of postmenopausal women with schizophrenia [57]. In the era of precision medicine, personalized treatments are recommended. This may include the use of safe and effective antipsychotic medications and the coadjuvant use of sexual hormones or SERMs. Insomnia is frequent in people with schizophrenia, and treatment needs to be tailored to the individual cases of patients. Postmenopausal women with schizophrenia potentially suffer from higher rates of insomnia and other sleep disturbances as a result of the universal condition of menopause.

The categorization of insomnia into primary and secondary conditions has been extensively described in the scientific literature. Although sleep disturbances have not been considered a core feature of schizophrenia, the clinical relevance of sleep has been highlighted by several authors [58]. Patients with schizophrenia often have a comorbid sleep disorder, such as obstructive sleep apnea, restless leg syndrome, or insomnia. Primary insomnia without other psychopathological symptoms is associated with worsening psychotic symptoms and a decrease in the quality of life [58]. Early treatment is crucial and is focused on the primary basis. In our review, the included studies did not differentiate between primary and secondary insomnia in the context of postmenopausal women [59]. Sleep problems are frequent in women at the time of menopause, and they are more frequent in those suffering from schizophrenia. After menopause, some sleep complaints are comorbid with depressive disorders, a fact that suggests that treating depressive symptoms may be sufficient to improve secondary insomnia. In our review, the effects of melatonin, sex hormones, and raloxifene were reported as adjunctive to the treatment with antipsychotic medications. Hormone treatment in monotherapy was not considered in the population of women with schizophrenia.

Other biological pathways should also be investigated in postmenopausal women with schizophrenia, for instance, the adrenaline–noradrenaline balance. Kornstein et al. [60] explored the efficacy of desvenlafaxine in perimenopausal and postmenopausal women diagnosed with major depressive disorder, and they found significant improvements in the Sheehan Disability Scale scores and the Menopause Rating Scale scores, suggesting that desvenlafaxine has efficacy in both peri- and postmenopausal women [60]. The interaction between the neurotransmitter systems and the hormone pathways should also be tested in women with schizophrenia.

This review has several limitations and strengths that should be clarified. The first limitation is that it is a narrative review rather than a systematic review. It is a nonsystematic narrative critical review of the literature on the topic of the effects of melatonin and the sex hormones on insomnia in postmenopausal women with schizophrenia, rather than an attempt to provide an all-encompassing review of the literature. This method allowed us to include papers in the field that have helped to answer the three questions we addressed at the beginning of the review. Another limitation of our review is that we could not separate primary and secondary insomnia in the included studies. The papers focusing on postmenopausal women with schizophrenia do not adequately describe insomnia according to these categorizations. However, melatonin and other sex hormone compounds were administered as adjunctive treatments. Despite the limitations we mention, the present study has several strengths. This is the first review integrating the use of melatonin and sex hormones, together, as potential treatments for women with schizophrenia in the postmenopausal stages. To the best of our knowledge, this is the first review addressing this issue in this specific population of women.

## 5. Conclusions

A large amount of research indicates that sleep problems are prevalent and severe in patients with schizophrenia. In our review, we focused our search on the use of melatonin and sex hormones for the treatment of insomnia in postmenopausal women with schizophrenia. Melatonin treatment improves the quality and efficiency of the sleep of patients with schizophrenia, compared to treatment with a placebo. However, to the best of our knowledge, there were no specific mentions with regard to the use of melatonin in postmenopausal women with schizophrenia, who are our target population.

Estrogen and progesterone pathways are dysregulated in major psychiatric disorders, such as in schizophrenia. However, the effects of estradiol and other similar compounds on sleep disorders in postmenopausal women with schizophrenia has not been sufficiently investigated. Raloxifene, a selective estrogen receptor modulator, has been found to exert positive effects on sleep disorders in healthy postmenopausal women. Although raloxifene may be effective for treating psychotic symptoms in postmenopausal women with schizophrenia, the specific effects on the sleep of postmenopausal schizophrenia populations is still understudied. Future studies should investigate the effectiveness of hormonal compounds on insomnia in postmenopausal women with schizophrenia.

## Figures and Tables

**Table 1:** Main characteristics of the most representative studies on the use of melatonin for insomnia.

Author and Year of Publication	Study Design	Sample N	Comparison Groups	Assessment	Results
Mishra A et al., 2020 [33]	Randomized rater-blinded	120 (49 F; 71 M)	Ramelteon plus risperidone or haloperidol, and control group receiving haloperidol or risperidone.	Baseline serum melatonin; serum AANAT; urinary melatonin; Pittsburgh Sleep Quality Index (PSQI).	Increase in night-time melatonin levels, AANAT serum, and urinary melatonin, and decrease in PSQI scores in ramelteon group.
Suresh Kumar et al., 2007 [34]	Randomised, double-blind	40 (13 F; 27 M)	Melatonin (3–12 mg) and placebo.	15-item structured questionnaire about sleep functioning.	Melatonin improved the sleep functioning better than placebo.
Shamir E et al., 2000 [35]	Randomised, double-blind, cross-over	19 (7 F; 12 M)	Controlled-release melatonin (2 mg) and placebo.	Activity- and rest-derived sleep/awake episodes.	Melatonin improved sleep efficiency and increased sleep duration.

**Table 2:** Estradiol as a potential target to treat insomnia in postmenopausal women with schizophrenia.

Animal Studies	
Estrogens are neuroprotective and show a positive influence on behavioral symptoms in animal models of schizophrenia (e.g., prepulse inhibition in rats).	Estrogens may influence sleep regulation.
**Human Studies**	
Estrogens may vary according to the phases of the menstrual cycle. High estrogen levels are associated with improvements in psychotic symptoms.	Estrogens and progesterone differ according to the phases of the menstrual cycle in premenopausal women and are found to influence sleep.
Sleep architecture worsens during the menopausal transition. Insomnia is frequent at menopause.	Loss of estrogens is associated with sleep disturbances at menopause. In schizophrenic women, this association may be stronger.

**Table 3:** Effects of sex hormones and raloxifene for the treatment of insomnia in postmenopausal schizophrenic patients.

Hormone	Hypothesis	Findings
Estradiol	Estradiol is capable of preventing dopamine D1/D2-receptor-mediated disruptions of sensorimotor gating in animal models.	Is estradiol a potential target for the treatment of insomnia? No results for postmenopausal populations.
Progesterone	Brexanolone, a synthetic allopregnanolone, prevents depression-like behaviors in animal models.	The psychotropic properties of progesterone have not been evaluated in postmenopausal schizophrenic patients.
Testosterone	High testosterone–estradiol ratio at menopause. Testosterone implicated in physiopathology of schizophrenia.	The use of testosterone to treat insomnia has not been evaluated.
Dehydroepiandrosterone (DHEA)	Precursors of androgens in women may be effective for the treatment of insomnia at menopause.	Potentially effective for the treatment of psychotic or cognitive symptoms; no results for insomnia.
Raloxifene (SERMs)	Positive effect on sleep in healthy women.	Potential effectiveness in postmenopausal schizophrenia. Future studies should consider insomnia as a primary outcome.

## Data Availability

The data presented in this review are available upon request from the corresponding author.

## References

[1] Marder S.R., Cannon T.D. (2019). Schizophrenia. N. Engl. J. Med..

[2] GBD 2017 Disease and Injury Incidence and Prevalence Collaborators (2018). Global, regional, and national incidence, prevalence, and years lived with disability for 354 diseases and injuries for 195 countries and territories, 1990–2017: A systematic analysis for the Global Burden of Disease Study 2017. Lancet.

[3] McCutcheon R.A., Krystal J.H., Howes O.D. (2020). Dopamine and glutamate in schizophrenia: Biology, symptoms and treatment. World Psychiatry.

[4] Reeve S., Sheaves B., Freeman D. (2019). Sleep Disorders in Early Psychosis: Incidence, Severity, and Association with Clinical Symptoms. Schizophr. Bull..

[5] Waite F., Myers E., Harvey A.G., Espie C.A., Startup H., Sheaves B., Freeman D. (2016). Treating Sleep Problems in Patients with Schizophrenia. Behav. Cogn. Psychother..

[6] Li S.X., Lam S.P., Zhang J., Yu M.W., Chan J.W., Chan C.S., Espie C.A., Freeman D., Mason O., Wing Y.K. (2016). Sleep Disturbances and Suicide Risk in an 8-Year Longitudinal Study of Schizophrenia-Spectrum Disorders. Sleep.

[7] Miller B.J., Parker C.B., Rapaport M.H., Buckley P.F., McCall W.V. (2019). Insomnia and suicidal ideation in nonaffective psychosis. Sleep.

[8] Stummer L., Markovic M., Maroney M.E. (2018). Pharmacologic Treatment Options for Insomnia in Patients with Schizophrenia. Medicines.

[9] Monti J.M., BaHammam A.S., Pandi-Perumal S.R., Bromundt V., Spence D.W., Cardinali D.P., Brown G.M. (2013). Sleep and circadian rhythm dysregulation in schizophrenia. Prog. Neuropsychopharmacol. Biol. Psychiatry.

[10] Park H.J., Park J.K., Kim S.K., Cho A.R., Kim J.W., Yim S.V., Chung J.H. (2011). Association of polymorphism in the promoter of the melatonin receptor 1A gene with schizophrenia and with insomnia symptoms in schizophrenia patients. J. Mol. Neurosci..

[11] Afonso P., Figueira M.L., Paiva T. (2011). Sleep-promoting action of the endogenous melatonin in schizophrenia compared to healthy controls. Int. J. Psychiatry Clin. Pract..

[12] Bersani G., Mameli M., Garavini A., Pancheri P., Nordio M. (2003). Reduction of night/day difference in melatonin blood levels as a possible disease-related index in schizophrenia. Neuro Endocrinol. Lett..

[13] Shirayama Y., Takahashi M., Suzuki M., Tsuruoka Y., Sato K. (2014). Effects of Add-on Ramelteon on Cognitive Impairment in Patients with Schizophrenia: An Open-label Pilot Trial. Clin. Psychopharmacol. Neurosci..

[14] Thomas N., Gurvich C., Hudaib A.R., Gavrilidis E., Kulkarni J. (2019). Dissecting the syndrome of schizophrenia: Associations between symptomatology and hormone levels in women with schizophrenia. Psychiatry Res..

[15] Thomas N., Gurvich C., Hudaib A.R., Gavrilidis E., de Castella R.A., Thomas E.H., Kulkarni J. (2021). Serum estradiol as a blood-based biomarker predicting hormonal treatment outcomes in women with schizophrenia. Psychoneuroendocrinology.

[16] Huang W., Li Y.H., Huang S.Q., Chen H., Li Z.F., Li X.X., Li X.S., Cheng Y. (2021). Serum Progesterone and Testosterone Levels in Schizophrenia Patients at Different Stages of Treatment. J. Mol. Neurosci..

[17] Gogos A., Ney L.J., Seymour N., Van Rheenen T.E., Felmingham K.L. (2019). Sex differences in schizophrenia, bipolar disorder, and post-traumatic stress disorder: Are gonadal hormones the link?. Br. J. Pharmacol..

[18] Buoli M., Caldiroli A., Serati M., Grassi S., Altamura A.C. (2016). Sex Steroids and Major Psychoses: Which Role for DHEA-S and Progesterone. Neuropsychobiology.

[19] Ray P., Mandal N., Sinha V.K. (2020). Change of symptoms of schizophrenia across phases of menstrual cycle. Arch. Womens Ment. Health.

[20] Van den Buuse M., Mingon R.L., Gogos A. (2015). Chronic estrogen and progesterone treatment inhibits ketamine-induced disruption of prepulse inhibition in rats. Neurosci. Lett..

[21] Frye C.A., Sora I. (2010). Progesterone reduces hyperactivity of female and male dopamine transporter knockout mice. Behav. Brain Res..

[22] Gogos A., van den Buuse M. (2015). Comparing the effects of 17β-oestradiol and the selective oestrogen receptor modulators, raloxifene and tamoxifen, on prepulse inhibition in female rats. Schizophr. Res..

[23] González-Rodríguez A., Bernardo M., Penadés R., Arias B., Ruiz Cortés V., Seeman M.V., Catalán R. (2017). Do FSH/LH ratio and gonadal hormone levels predict clinical improvement in postmenopausal schizophrenia women?. Arch. Womens Ment. Health.

[24] Schaedel Z., Holloway D., Bruce D., Rymer J. (2021). Management of sleep disorders in the menopausal transition. Post. Reprod. Health.

[25] Caretto M., Giannini A., Simoncini T. (2019). An integrated approach to diagnosing and managing sleep disorders in menopausal women. Maturitas.

[26] Proserpio P., Marra S., Campana C., Agostoni E.C., Palagini L., Nobili L., Nappi R.E. (2020). Insomnia and menopause: A narrative review on mechanisms and treatments. Climacteric.

[27] Duan C., Jenkins Z.M., Castle D. (2021). Therapeutic use of melatonin in schizophrenia: A systematic review. World J. Psychiatry.

[28] Borba C.P., Fan X., Copeland P.M., Paiva A., Freudenreich O., Henderson D.C. (2011). Placebo-controlled pilot study of ramelteon for adiposity and lipids in patients with schizophrenia. J. Clin. Psychopharmacol..

[29] Sahbaz C., Özer O.F., Kurtulmus A., Kırpınar I., Sahin F., Guloksuz S. (2019). Evidence for an association of serum melatonin concentrations with recognition and circadian preferences in patients with schizophrenia. Metab. Brain. Dis..

[30] Maiti R., Mishra B.R., Jena M., Mishra A., Nath S. (2021). Effect of Haloperidol and Risperidone on Serum Melatonin and GAP-43 in Patients with Schizophrenia: A Prospective Cohort Study. Clin. Psychopharmacol. Neurosci..

[31] Palagini L., Manni R., Aguglia E., Amore M., Brugnoli R., Bioulac S., Bourgin P., Micoulaud Franchi J.A., Girardi P., Grassi L. (2021). International Expert Opinions and Recommendations on the Use of Melatonin in the Treatment of Insomnia and Circadian Sleep Disturbances in Adult Neuropsychiatric Disorders. Front. Psychiatry.

[32] Geoffroy P.A., Micoulaud Franchi J.A., Lopez R., Schroder C.M., Membres du Consensus Mélatonine SFRMS (2019). The use of melatonin in adult psychiatric disorders: Expert recommendations by the French institute of medical research on sleep (SFRMS). Encephale.

[33] Mishra A., Maiti R., Mishra B.R., Jena M., Nath S., Sahu P. (2020). Effect of add-on ramelteon therapy on sleep and circadian rhythm disruption in patients with schizophrenia: A randomized controlled trial. Eur. Neuropsychopharmacol..

[34] Suresh Kumar P.N., Andrade C., Bhakta S.G., Singh N.M. (2007). Melatonin in schizophrenic outpatients with insomnia: A double-blind, placebo-controlled study. J. Clin. Psychiatry.

[35] Shamir E., Laudon M., Barak Y., Anis Y., Rotenberg V., Elizur A., Zisapel N. (2000). Melatonin improves sleep quality of patients with chronic schizophrenia. J. Clin. Psychiatry.

[36] Baandrup L., Lindschou J., Winkel P., Gluud C., Glenthoj B.Y. (2016). Prolonged-release melatonin versus placebo for benzodiazepine discontinuation in patients with schizophrenia or bipolar disorder: A randomised, placebo-controlled, blinded trial. World J. Biol. Psychiatry.

[37] Lord C., Sekerovic Z., Carrier J. (2014). Sleep regulation and sex hormones exposure in men and women across adulthood. Pathol. Biol..

[38] Bezerra A.G., Andersen M.L., Pires G.N., Tufik S., Hachul H. (2018). Effects of hormonal contraceptives on sleep—A possible treatment for insomnia in premenopausal women. Sleep Sci..

[39] Kalleinen N., Aittokallio J., Lampio L., Kaisti M., Polo-Kantola P., Polo O., Heinonen O.J., Saaresranta T. (2021). Sleep during menopausal transition: A 10-year follow-up. Sleep.

[40] Morssinkhof M.W.L., van Wylick D.W., Priester-Vink S., van der Werf Y.D., den Heijer M., van den Heuvel O.A., Broekman B.F.P. (2020). Associations between sex hormones, sleep problems and depression: A systematic review. Neurosci. Biobehav. Rev..

[41] Cai H., Zhou X., Dougherty G.G., Reddy R.D., Haas G.L., Montrose D.M., Keshavan M., Yao J.K. (2018). Pregnenolone-progesterone-allopregnanolone pathway as a potential therapeutic target in first-episode antipsychotic-naïve patients with schizophrenia. Psychoneuroendocrinology.

[42] Barak Y., Glue P. (2020). Progesterone loading as a strategy for treating postpartum depression. Hum. Psychopharmacol..

[43] Sander B., Muftah A., Sykes Tottenham L., Grummisch J.A., Gordon J.L. (2021). Testosterone and depressive symptoms during the late menopause transition. Biol. Sex Differ..

[44] Kianimehr G., Fatehi F., Hashempoor S., Khodaei-Ardakani M.R., Rezaei F., Nazari A., Kashani L., Akhondzadeh S. (2014). Raloxifene adjunctive therapy for postmenopausal women suffering from chronic schizophrenia: A randomized double-blind and placebo controlled trial. Daru.

[45] Natale V., Albertazzi P., Missiroli N., Pedrini D., Salgarello M. (2004). Effects of raloxifene on mood, sleep, libido and cognitive function in postmenopausal healthy women: A pilot study. Maturitas.

[46] Biri A., Korucuoglu U., Ilhan M.N., Ciftci B., Bozkurt N., Guner H. (2009). Evaluation of the sexual function and quality of life in raloxifene treated postmenopausal women. Arch. Gynecol. Obstet..

[47] Zhu X.M., Zheng W., Li X.H., Cai D.B., Yang X.H., Ungvari G.S., Ng C.H., Wang X.P., Kulkarni J., Grigg J. (2018). Adjunctive raloxifene for postmenopausal women with schizophrenia: A meta-analysis of randomized, double-blind, placebo-controlled trials. Schizophr. Res..

[48] Wang Q., Dong X., Wang Y., Li X. (2018). Raloxifene as an adjunctive treatment for postmenopausal women with schizophrenia: A meta-analysis of randomized controlled trials. Arch. Womens Ment. Health.

[49] Andrabi S.S., Vishnoi S., Kaushik M., Parveen K., Tabassum H., Akram M., Parvez S. (2019). Reversal of Schizophrenia-like Symptoms and Cholinergic Alterations by Melatonin. Arch. Med. Res..

[50] Onaolapo A.Y., Aina O.A., Onaolapo O.J. (2017). Melatonin attenuates behavioural deficits and reduces brain oxidative stress in a rodent model of schizophrenia. Biomed. Pharmacother..

[51] Afonso A.C., Pacheco F.D., Canever L., Wessler P.G., Mastella G.A., Godoi A.K., Hubbe I., Bischoff L.M., Bialecki A.V.S., Zugno A.I. (2020). Schizophrenia-like behavior is not altered by melatonin supplementation in rodents. An. Acad. Bras. Cienc..

[52] Mahmoud G.S., El-Deek H.E. (2019). Melatonin modulates inflammatory mediators and improves olanzapine-induced hepatic steatosis in rat model of schizophrenia. Int. J. Physiol. Pathophysiol. Pharmacol..

[53] Igwe S.C., Brigo F. (2018). Does Melatonin and Melatonin Agonists Improve the Metabolic Side Effects of Atypical Antipsychotics?: A Systematic Review and Meta-analysis of Randomized Controlled Trials. Clin. Psychopharmacol. Neurosci..

[54] Oliveira P., Coroa M., Madeira N. (2019). Treatment Options for Insomnia in Schizophrenia: A Systematic Review. Pharmacopsychiatry.

[55] Arad M., Weiner I. (2010). Contrasting effects of increased and decreased dopamine transmission on latent inhibition in ovariectomized rats and their modulation by 17beta-estradiol: An animal model of menopausal psychosis?. Neuropsychopharmacology.

[56] González-Rodríguez A., Seeman M.V. (2018). Pharmacotherapy for schizophrenia in postmenopausal women. Expert. Opin. Pharmacother..

[57] Labad J., Cobo J., Núñez C., Creus M., García-Parés G., Cuadras D., Franco J., Miquel E., Reyes J.C., Marcó-García S. (2020). Effects of raloxifene on cognition in postmenopausal women with schizophrenia: A 24-week double-blind, randomized, parallel, placebo-controlled trial. Eur. Arch. Psychiatry Clin. Neurosci..

[58] Kaskie R.E., Graziano B., Ferrarelli F. (2017). Schizophrenia and sleep disorders: Links, risks, and management challenges. Nat. Sci. Sleep.

[59] Baker F.C., de Zambotti M., Colrain I.M., Bei B. (2018). Sleep problems during the menopausal transition: Prevalence, impact, and management challenges. Nat. Sci. Sleep.

[60] Kornstein S.G., Clayton A.H., Bao W., Guico-Pabia C.J. (2015). A pooled analysis of the efficacy of desvenlafaxine for the treatment of major depressive disorder in perimenopausal and postmenopausal women. J. Womens Health.

